# Evaluation of Clinical Practice Guidelines on Fall Prevention and Management for Older Adults: A Systematic Review

**DOI:** 10.1001/jamanetworkopen.2021.38911

**Published:** 2021-12-15

**Authors:** Manuel M. Montero-Odasso, Nellie Kamkar, Frederico Pieruccini-Faria, Abdelhady Osman, Yanina Sarquis-Adamson, Jacqueline Close, David B. Hogan, Susan Winifred Hunter, Rose Anne Kenny, Lewis A. Lipsitz, Stephen R. Lord, Kenneth M. Madden, Mirko Petrovic, Jesper Ryg, Mark Speechley, Munira Sultana, Maw Pin Tan, N. van der Velde, Joe Verghese, Tahir Masud

**Affiliations:** 1 Schulich School of Medicine and Dentistry, Division of Geriatric Medicine, Department of Medicine, The University of Western Ontario, London, Ontario, Canada; 2 Gait and Brain Lab, Parkwood Institute, Lawson Health Research Institute, London, Ontario, Canada; 3 Department of Epidemiology and Biostatistics, The University of Western Ontario, London, Ontario, Canada; 4 Falls, Balance and Injury Research Centre, Neuroscience Research Australia, University of New South Wales, Sydney, Australia; 5 Prince of Wales Clinical School, Medicine, University of New South Wales, Sydney, Australia; 6 Division of Geriatric Medicine, Department of Medicine, Cumming School of Medicine, University of Calgary, Calgary, Alberta, Canada; 7 Faculty of Health Sciences, University of Western Ontario, London, Ontario, Canada; 8 School of Physical Therapy, University of Western Ontario, London, Ontario, Canada; 9 Department of Medical Gerontology, Mercers Institute for Ageing, St James Hospital, Dublin, Ireland; 10 Hinda and Arthur Marcus Institute for Aging Research, Hebrew SeniorLife, Harvard Medical School, Boston, Massachusetts; 11 Falls, Balance and Injury Research Centre, Neuroscience Research Australia, Sydney, New South Wales, Australia; 12 School of Public Health and Community Medicine, University of New South Wales, Sydney, New South Wales, Australia; 13 Division of Geriatric Medicine, Department of Medicine, Department of Internal Medicine, Section of Geriatric Medicine, University of British Columbia, Vancouver, British Columbia, Canada; 14 Division of Geriatric Medicine, Department of Medicine, Faculty of Medicine, University of British Columbia, Vancouver, British Columbia, Canada; 15 Section of Geriatrics, Department of Internal Medicine and Paediatrics, Faculty of Medicine and Health Sciences, Ghent University, Ghent, Belgium; 16 Department of Geriatric Medicine, Odense University Hospital, Odense, Denmark; 17 Geriatric Research Unit, Department of Clinical Research, University of Southern Denmark, Odense, Denmark; 18 Schulich Interfaculty Program in Public Health, Schulich School of Medicine & Dentistry, University of Western Ontario, London, Ontario, Canada; 19 Centre for Innovation in Medical Engineering, Faculty of Engineering, University of Malaysia, Kuala Lumpur, Malaysia; 20 Department of Medicine, Faculty of Medicine, University of Malaya, Kuala Lumpur, Malaysia; 21 Section of Geriatric Medicine, Department of Internal Medicine, Amsterdam Public Health, Academic Medical Center, University of Amsterdam, Amsterdam, the Netherlands; 22 Institute for Aging Research, Department of Medicine, Albert Einstein College of Medicine, Bronx, New York; 23 Department of Neurology, Albert Einstein College of Medicine, Bronx, New York; 24 Department of Geriatric Medicine, Nottingham University Hospitals NHS Trust, Nottingham, United Kingdom

## Abstract

**Importance:**

With the global population aging, falls and fall-related injuries are ubiquitous, and several clinical practice guidelines for falls prevention and management for individuals 60 years or older have been developed. A systematic evaluation of the recommendations and agreement level is lacking.

**Objectives:**

To perform a systematic review of clinical practice guidelines for falls prevention and management for adults 60 years or older in all settings (eg, community, acute care, and nursing homes), evaluate agreement in recommendations, and identify potential gaps.

**Evidence Review:**

A systematic review following Preferred Reporting Items for Systematic Reviews and Meta-analyses statement methods for clinical practice guidelines on fall prevention and management for older adults was conducted (updated July 1, 2021) using MEDLINE, PubMed, PsycINFO, Embase, CINAHL, the Cochrane Library, PEDro, and Epistemonikos databases. Medical Subject Headings search terms were related to falls, clinical practice guidelines, management and prevention, and older adults, with no restrictions on date, language, or setting for inclusion. Three independent reviewers selected records for full-text examination if they followed evidence- and consensus-based processes and assessed the quality of the guidelines using Appraisal of Guidelines for Research & Evaluation II (AGREE-II) criteria. The strength of the recommendations was evaluated using Grades of Recommendation, Assessment, Development, and Evaluation scores, and agreement across topic areas was assessed using the Fleiss κ statistic.

**Findings:**

Of 11 414 records identified, 159 were fully reviewed and assessed for eligibility, and 15 were included. All 15 selected guidelines had high-quality AGREE-II total scores (mean [SD], 80.1% [5.6%]), although individual quality domain scores for clinical applicability (mean [SD], 63.4% [11.4%]) and stakeholder (clinicians, patients, or caregivers) involvement (mean [SD], 76.3% [9.0%]) were lower. A total of 198 recommendations covering 16 topic areas in 15 guidelines were identified after screening 4767 abstracts that proceeded to 159 full texts. Most (≥11) guidelines strongly recommended performing risk stratification, assessment tests for gait and balance, fracture and osteoporosis management, multifactorial interventions, medication review, exercise promotion, environment modification, vision and footwear correction, referral to physiotherapy, and cardiovascular interventions. The strengths of the recommendations were inconsistent for vitamin D supplementation, addressing cognitive factors, and falls prevention education. Recommendations on use of hip protectors and digital technology or wearables were often missing. None of the examined guidelines included a patient or caregiver panel in their deliberations.

**Conclusions and Relevance:**

This systematic review found that current clinical practice guidelines on fall prevention and management for older adults showed a high degree of agreement in several areas in which strong recommendations were made, whereas other topic areas did not achieve this level of consensus or coverage. Future guidelines should address clinical applicability of their recommendations and include perspectives of patients and other stakeholders.

## Key Points

**Question:**

What are the most common consistent recommendations in fall prevention clinical practice guidelines, across settings, for adults 60 years or older?

**Findings:**

In this systematic review of 198 recommendations across 15 selected guidelines, most guidelines recommended fall risk stratification, assessment tools, fractures or osteoporosis management, multifactorial interventions, medication review, exercise, physiotherapy referral, environment modification, and vison, footwear, and cardiovascular interventions. Recommendations on vitamin D supplementation, addressing cognitive factors, and education were inconsistent, whereas hip protectors, digital technology, clinical applicability, and stakeholder involvement were less commonly addressed.

**Meaning:**

This systematic review found that agreement was high on several recommendations for fall prevention clinical practice guidelines for older adults, but certain areas, including stakeholder perspectives and clinical applicability, were often not addressed.

## Teaser

This systematic review assesses current recommended clinical practice guidelines, evaluating agreement among the most common recommendations and highlighting potential gaps, for falls prevention and management across community, acute care, and nursing home settings among older adults.

## Introduction

Falls and fall-related injuries are common for older adults,^1^ with approximately 30% of adults 60 years of age or older falling each year.^2,3,4^ Falls are more likely for older adults with greater frailty severity and among those living in nursing homes.^5,6^ Consequences of falls include injuries,^7^ fractures,^8^ problems with mobility, depression, loss of independence,^9,10^ and a substantial economic burden on health care systems.^11^

Falls and their concomitant injuries represent a worldwide phenomenon.^12^ Accordingly, several medical societies and organizations in different countries have created clinical practice guidelines for fall prevention and management.^13,14,15,16,17,18,19,20,21,22,23,24,25,26,27^ These guidelines are typically based on systematic reviews of the available evidence and consensus by experts in the fields of geriatric medicine, rehabilitation medicine, and physiotherapy, among others.^28,29^ Although several of these clinical practice guidelines for fall prevention have been published, little is known about the level of agreement between the recommendations made by them. Clinicians face the challenge of selecting high-quality guidelines based on robust methods with internally and externally validated recommendations applicable to their setting in informing their practice.^30,31^

We aimed to (1) systematically review existing clinical practice guidelines on fall prevention and management for older adults; (2) identify common areas evaluated and level of agreement in the recommendations made; (3) address fall risk stratification in each guideline, describing which assessments are recommended to guide this and inform management across settings (eg, community, acute care, and nursing homes); and (4) identify potential gaps and areas that should be addressed in future clinical practice guidelines.

## Methods

We followed the Preferred Reporting Items for Systematic Reviews and Meta-analyses (PRISMA) reporting guideline and preregistered in PROSPERO (CRD42020173597). This systematic review was performed under the umbrella of the World Falls Guidelines for Prevention and Management of Falls in Older Adults.^32^

### Identification of Guidelines

Our initial search on April 2, 2020, was updated July 1, 2021, and included the following databases: MEDLINE, PubMed, PsycINFO, Embase, CINAHL (Cumulative Index to Nursing and Allied Health Literature), the Cochrane Library, PEDro (Physiotherapy Evidence Database), and Epistemonikos. Three of us (M.M.M.-O., S.W.H., and T.M.) also provided consultation to include guidelines potentially not indexed in databases.

### Search Terms

Our search used Medical Subject Headings terms pertaining to (1) falls, (2) clinical practice guidelines, (3) management and prevention, and (4) older adults (eTable 1 in Supplement 1 describes our search syntax).

### Inclusion Criteria

The inclusion criteria were (1) outcome of guidelines: fall reduction, prevention, and management; (2) study type: clinical practice guidelines for preventing or managing falls categorized as consensus- or evidence-based guidelines^13^; and (3) target population of guidelines: older adults. There were no restrictions on date, language, or setting for inclusion.

### Screening, Review Process, and Quality Assessment

Three of us as independent reviewers (M.M.M.-O., N.K., and Y.S.-A.) selected records for full-text examination if they followed evidence- and consensus-based processes; disagreements were resolved by consensus. Three of us as reviewers (N.K., F.P.-F., and A.O.) assessed guideline quality using the 23-item Appraisal of Guidelines for Research & Evaluation II (AGREE-II) tool^31^ (eTable 2 in Supplement 1). The scores for AGREE-II range from 0 to 100, with higher scores indicating higher quality. Extracted recommendations were grouped in common areas and independently appraised by 3 of us (N.K., F.P.-F., and A.O.; blinded among us 3) using Grades of Recommendation, Assessment, Development, and Evaluation (GRADE),^29,33^ which reflects the strength of the recommendation (1 = strong; 2 = weak) paired with the quality of the supporting evidence (A = high; B = moderate; and C = low). Agreement across guidelines for specific recommendations was assessed using the Fleiss κ statistic.

## Results

**Figure 1.**
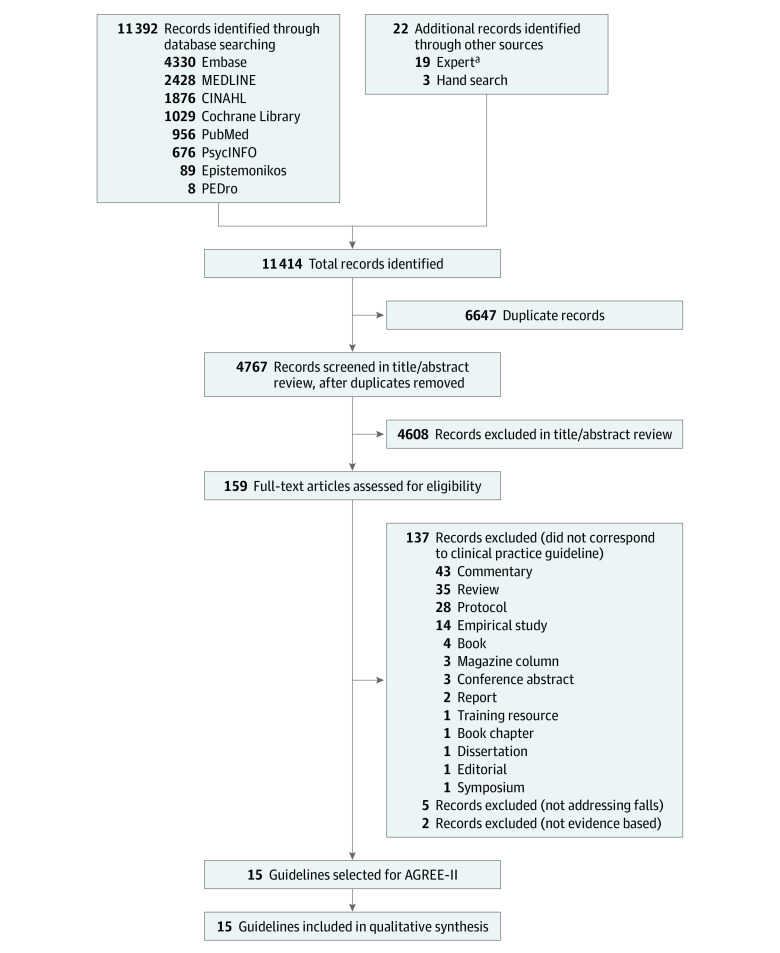
Preferred Reporting Items for Systematic Reviews and Meta-analyses Flowchart of Search Yield AGREE-II indicates Appraisal of Guidelines for Research & Evaluation II. ^a^Records suggested by 3 of us who are experts in the field of geriatric medicine (M.M.M.-O., S.R.L., and T.M.).

Our search yielded 11 414 records. There were 6647 duplicates, and 4608 records were excluded after title and abstract review, resulting in 159 records that were fully reviewed and assessed for eligibility (Figure 1).^34^ Of the 159 records, 144 were excluded, yielding 15 records retained for final analyses and included in the data synthesis.^13,14,15,16,17,18,19,20,21,22,23,24,25,26,27^ Table 1 shows the quality assessment characteristics using the AGREE-II tool for the 15 guidelines selected.

**Table 1.** Quality Assessment Total and Domain-Specific Scores of the Guidelines Using AGREE-II^a^

Source or guideline	AGREE-II total score, %	AGREE-II domain scores, %					
		1: Scope and purpose	2: Stakeholder involvement	3: Rigor of development	4: Clarity of presentation	5: Applicability	6: Editorial independence
Baraff et al,^16^ 1997 (US)	78.5	96.3	81.5	72.2	88.9	62.5	88.9
AGILE,^21^ 1998 (UK)	74.6	94.4	68.5	66.7	94.4	56.9	91.7
Feder et al,^19^ 2000 (UK)	77.8	92.6	66.7	81.9	81.5	54.2	97.2
AGS/BGS/AAOS,^13^ 2001 (US/UK)	84.5	94.4	81.5	82.6	96.3	66.7	100
Moreland et al,^24^ 2003 (Canada)	80.0	96.3	68.5	80.6	83.3	66.7	91.7
ACSQHC,^15^ 2009 (Australia)	81.9	94.4	83.3	68.8	94.4	79.2	100
FSGG,^17^ 2011 (France)	78.0	92.6	74.1	75.0	85.2	59.7	100
NICE,^18^ 2013 (UK)	92.8	98.1	94.4	91.7	92.6	86.1	100
STEADI,^27^ 2013 (US)	74.2	90.7	81.5	66.7	81.5	58.3	88.9
Jung et al,^22^ 2014 (Korea)	77.5	90.7	64.8	77.8	90.7	56.9	97.2
RACGP,^26^ 2016 (Australia)	69.7	83.3	72.2	58.3	83.3	54.2	100
KAIM/KGS,^23^ 2017 (Korea)	80.4	79.6	81.5	86.8	90.7	51.4	97.2
RNAO,^25^ 2017 (Canada)	88.0	94.4	86.1	84.4	88.9	85.4	100
SENATOR ONTOP,^20^ 2017 (Ireland)	79.2	94.4	61.1	86.1	85.2	52.8	100
USPSTF,^14^ 2018 (US)	82.9	92.6	79.6	82.6	96.3	59.7	100
Mean (SD) [range]	80.1 (5.6) [69.7-92.8]	92.3 (4.8) [79.6-98.1]	76.3 (9.0) [61.1-94.4]	77.6 (9.3) [58.3-91.7]	88.7 (5.4) [81.5-96.3]	63.4 (11.4) [51.4-86.1]	96.9 (4.2) [88.9-100]

Abbreviations: ACSQHC, Australian Commission on Safety and Quality in Health Care; AGILE, a recognized professional network of the Chartered Society of Physiotherapy; AGREE-II, Appraisal of Guidelines for Research & Evaluation–II; AGS/BGS/AAOS, American Geriatrics Society/British Geriatrics Society/American Academy of Orthopaedic Surgeons; FSGG, French Society of Geriatrics and Gerontology; KAIM/KGS, Korean Association of Internal Medicine/Korean Geriatrics Society; NICE, National Institute for Health and Care Excellence; RACGP, The Royal Australian College of General Practitioners; RNAO, Registered Nurses’ Association of Ontario; SENATOR ONTOP, software engine for the assessment & optimization of drug and non-drug therapy—older persons optimal evidence-based non-drug therapies in older people; STEADI, Stopping Elderly Accidents, Deaths and Injuries; USPSTF, US Preventive Services Task Force.

^a^ Scores range from 0 to 100, with higher scores indicating higher quality.

### Quality Assessment

**Figure 2.**
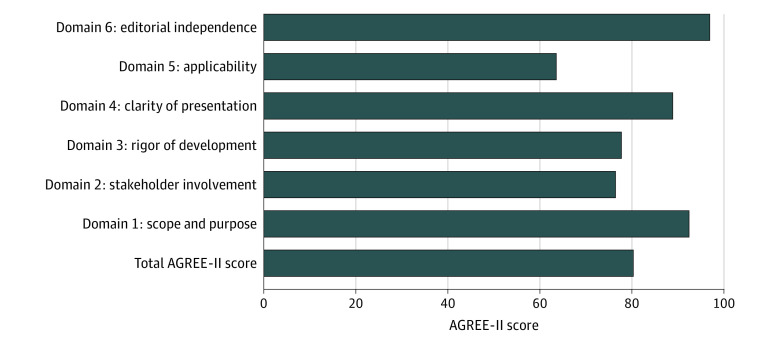
Mean Appraisal of Guidelines for Research & Evaluation II (AGREE-II) Total and Domain-Specific Scores Across Guidelines^31^

The AGREE-II total scores were high across all guidelines (mean [SD], 80.1% [5.6%]; range, 69.7%-92.8%). Descriptive statistics for AGREE-II scores by domain are given in Table 1, and mean AGREE-II scores by domain are illustrated in Figure 2. Domain 6 (editorial independence, competing interests, and conflicts of interest disclosed) scored highest across guidelines. Domain 1 (guideline objectives, clinical research question being addressed, and target population) and domain 4 (clarity of presentation) also showed high mean scores. Domain 2 (representation and involvement from professional backgrounds, and by patients and stakeholders) showed moderate mean scores (mean [SD], 76.3% [9.0%]) mainly owing to involvement of only clinicians in some of the guidelines; however, none of guidelines included an exclusive panel of patients or caregivers involved in the entirety of the guideline development process. Domain 3 (systematic methods used to obtain evidence, strengths and limitations clearly outlined, and the extent to which the health benefits and adverse effects of each recommendation are considered) scores were moderately high with more variability. Domain 5 (applicability of the recommendations, descriptors of facilitators and barriers to the application of each recommendation, and advice on tools and resources for applying each recommendation) scored consistently lower (mean [SD], 63.4% [11.4%]) than the other domains mainly because only 5 guidelines provided a toolkit or a step-by-step process in how to apply the recommendations.^15,18,21,24,25^

### Recommendations and Agreement Across Guidelines

After screening 4767 abstracts that proceeded to 159 full texts, we extracted 198 recommendations from the 15 guidelines that we grouped into 16 commonly addressed topic areas (Table 2). Topic areas that were presented in more than 40% of the guidelines were included in Table 2. Each topic area in Table 2 includes an accompanying GRADE score, which reflects the strength of the recommendation and the quality of the evidence. Across all areas and in all guidelines, the direction of the recommendation was in favor of the guideline (rather than recommending against its use). For definitions of the 16 commonly addressed topic areas, refer to eTable 3 in Supplement 1. The following topic areas were presented in less than 40% of the guidelines: addressing the use of canes or walking aids in the recommendations, alcohol use, depression, urinary incontinence, hearing impairment, atypical blood glucose, social isolation, and functional dependence as risk factors for falls, followed by staff education in nursing homes as part of interventions to prevent and manage falls.

**Table 2.** Guidelines Appraised With Evidence and Strength for Each Recommendation Stratified by Topic Areas Identified

16 Areas identified	No. (%) of guidelines addressing this area	Mode of GRADE score^a^	GRADE agreement Fleiss *κ*	15 Guidelines included^a^														
				Baraff et al,^16^ 1997	AGILE,^21^ 1998	Feder et al,^19^ 2000	AGS/BGS/AAOS,^13^ 2001	Moreland et al,^24^ 2003	ACSQHC,^15^ 2009	FSGG,^17^ 2011	NICE,^18^ 2013	STEADI,^27^ 2013	Jung et al,^22^ 2014	RACGP,^26^ 2016	KAIM/KGS,^23^ 2017	RNAO,^25^ 2017	SENATOR ONTOP,^20^ 2017	USPSTF,^14^ 2018
Risk stratification	13 (87)	1A	.92	NA	1A	1A	1A	1A	1A	1C	1B	1A	1A	1A	1C	1A	NA	1A
Assessment tools	15 (100)	1A	.88	1A	1A	1B	1A	1A	1A	1C	1A	1A	1A	1A	1C	1B	1B	1A
Fractures and osteoporosis management	11 (73)	1A	.83	1A	1A	NA	NA	NA	1A	1C	1A	1A	2B	1A	1A	1C	NA	1A
Multifactorial interventions	14 (93)	1A	.82	NA	1A	1A	2B	1B	1A	1C	1A	1C	1A	1A	1A	1A	1C	1C
Medication review	14 (93)	1A	.68	1A	1B	NA	1C	1A	1B	1C	1A	1A	1A	1A	1C	1A	1B	2C
Exercise interventions	15 (100)	1A	.88	1A	1A	1B	1B	1B	1A	1C	1A	1B	1A	1A	1A	1A	1B	1B
Vitamin D supplementation	11 (73)	Mixed	.30	1A	NA	NA	2C	NA	1A	1C	2C	1A	1A	1A	2C	1B	NA	2C
Hip protectors	9 (60)	Underrep	.69	NA	NA	1B	1C	NA	1A	2C	2B	1A	1A	NA	NA	1B	NA	1A
Vision modification	13 (87)	1B	.66	1A	NA	NA	1C	1B	2B	1C	1B	1A	1A	1A	1C	2C	1B	1B
Environment modification	14 (93)	1A	.70	1B	1A	1B	1B	1A	1A	1C	1A	1A	1A	1A	1C	1A	NA	2C
Cognitive factors management	11 (73)	Mixed	.39	1B	NA	NA	1B	1B	1C	1C	2C	1B	1A	1A	NA	2C	NA	2C
Physiotherapy referral	13 (87)	1A	.50	1A	2B	NA	1B	1B	1A	1C	1B	1A	1A	NA	1A	2C	1C	2B
Falls education	12 (80)	Mixed	.20	NA	2C	2A	2B	2C	1A	1C	1B	1B	1A	NA	NA	1A	2B	2B
Cardiovascular intervention	13 (87)	1B	.61	1B	2C	1B	1C	1B	1B	1C	1B	1B	1A	1A	1C	NA	NA	1C
Footwear evaluation and intervention	12 (80)	1A	.42	1B	2B	NA	2C	1B	2C	1C	1A	1A	NA	1A	1C	1A	NA	2C
Technology	7 (47)	Underrep	.78	NA	NA	NA	1C	1B	NA	NA	NA	1C	1A	NA	NA	2B	1B	1C
Areas addressed in each guideline (of 16), %				69	75	50	94	81	94	100	94	100	94	75	75	100	50	100
Setting of intended recommendations^b^																		
Community dwelling				✓	✓	✓	✓	✓	✓	✓	✓	✓		✓	✓	✓	✓	✓
Nursing homes					✓	✓	✓	✓			✓		✓		✓	✓	✓	
Acute care and hospitals					✓						✓				✓	✓	✓	

Abbreviations: ACSQHC, Australian Commission on Safety and Quality in Health Care; AGILE, a recognized professional network of the Chartered Society of Physiotherapy; AGS/BGS/AAOS, American Geriatrics Society/British Geriatrics Society/American Academy of Orthopaedic Surgeons; FSGG, French Society of Geriatrics and Gerontology; GRADE, Grades of Recommendation, Assessment, Development, and Evaluation; KAIM/KGS, Korean Association of Internal Medicine/Korean Geriatrics Society; NA, not available; NICE, National Institute for Health and Care Excellence; RACGP, The Royal Australian College of General Practitioners; RNAO, Registered Nurses’ Association of Ontario; SENATOR ONTOP, software engine for the assessment & optimization of drug and non-drug therapy—older persons optimal evidence-based non-drug therapies in older people; STEADI, Stopping Elderly Accidents, Deaths and Injuries; Underrep, Underrepresented; USPSTF, US Preventive Services Task Force.

^a^ GRADE strength of recommendation (1 = strong; 2 = weak) and quality of evidence (A = high quality; B = moderate quality; C = low quality).

^b^ The check mark indicates setting of intended recommendation.

Of 15 guidelines, 4 addressed all 16 topic areas identified,^14,17,25,27^ whereas 5 addressed at least 13 of them.^13,15,18,22,24^ Two topic areas (use of assessment tools for individuals who screened positive in falls risk, and exercise interventions) were covered in all of the guidelines, indicating consistent support for their importance. Medication review for fall risk–increasing drugs, use of multifactorial interventions to manage falls, and environment modification to prevent falls were recommended in 14 of the guidelines. Thirteen of the guidelines recommended performing risk stratification to detect high-risk individuals if they screened positive in the case-finding step using gait and balance tests. Thirteen guidelines also recommended conducting vision interventions, cardiovascular interventions for falls, and referral to a physiotherapist for exercises and balance retraining. Twelve guidelines recommended footwear evaluation and intervention and falls prevention education. Concerning the strength of the recommendations and quality of the evidence supporting the recommendation, GRADE A scores were most commonly found for 9 topic areas (Table 2). Agreement across guidelines was high (κ > 0.80) for 5 areas: risk stratification, assessment tools, fractures and osteoporosis management, exercise interventions, and use of multifactorial interventions. Agreement was moderate (κ = 0.50-0.80) for 7 topic areas and low (κ < 0.5) for the remaining 4 areas (Table 2).

### Inconsistent and Underrepresented Topic Areas in Recommendations Across Guidelines

Recommendations on vitamin D supplementation (κ = 0.30) and education on falls prevention (κ = 0.20) had low levels of agreement across the 15 guidelines. Seven guidelines strongly recommended the use of vitamin D supplementation, 4 guidelines provided weak recommendations, and the remaining 4 guidelines did not address the topic. For education on falls prevention, 6 guidelines provided strong recommendations to offer patients and caregivers education on fall prevention and management strategies, 6 gave weak recommendations, and 3 did not address this topic area. Recommendations for addressing cognitive impairment during fall risk assessment and management were present in 11 guidelines, with low agreement across them (κ = 0.39). Physiotherapy referral was recommended in 13 guidelines but with low agreement (κ = 0.50).

The use of hip protectors to prevent fall-related injuries and the use of digital technology (including wearables) to detect, prevent, or manage falls had a low level of agreement across the 15 guidelines. For recommendations on hip protectors, 7 guidelines provided strong recommendations for their use in nursing home settings, 2 guidelines provided weak recommendations, and 6 guidelines did not address their use. Recommendations on the use of digital technology had similar results. Six guidelines provided strong recommendations to use digital technology, 1 guideline provided a weak recommendation, and 8 guidelines did not address their use.

### Risk Stratification

Most guidelines strongly recommended risk stratification using “case finding” self-reported questions, including fall history, fear of falling, and gait and balance difficulties, and reserving gait and balance testing for those who screen positive on these questions. Five guidelines included a risk-stratification algorithm, but evidence validating the algorithm was not consistently presented, as described in Table 3.^2,3,4,13,14,15,16,17,18,19,20,21,22,23,24,25,26,27,35,36^ The majority of these algorithms followed the format proposed by the American Geriatrics Society/British Geriatrics Society/American Academy of Orthopaedic Surgeons (AGS/BGS/AAOS) guidelines.^13^ Individuals who had either no falls or 1 noninjurious fall in the last year and no impairment of balance and gait evident on examination were considered low risk, with a reassessment suggested sometime in the future. The interval proposed to reassess these low-risk individuals ranged from 1 year to 2 years across the guidelines examined.

**Table 3.** Description of Risk Stratification by Guidelines and Use of an Algorithm

Guidelines identified	Determination steps for risk assessment in the recommendations						Gaps and areas to expand
	Stratification method	Fall history	Age, y	Sex	Gait, balance, and mobility assessment	Other	
Baraff,^16^ 1997	Narrative	Any previous falls	NA	NA	TUG test	NA	Absence of a clear risk stratification methods algorithm
AGILE,^21^ 1998	NA	NA	NA	NA	NA	NA	
Feder et al,^19^ 2000	NA	NA	NA	NA	NA	NA	
AGS/BGS/AAOS,^2,3,4,13,35^ 2001	Algorithm	1 Fall in 12 mo: gait and balance evaluation; recurrent falls in 12 mo or an acute fall or difficulty with walking and balance: fall evaluation^1^	NA	NA	TUG test	NA	Individuals with no fall history or low (1 fall) fall history may fall and require fall evaluation^36^
Moreland et al,^24^ 2003	Narrative	NA	≥74	Female	Tinetti Performance-Oriented Assessments of Gait and Balance	NA	Fall history is not included in risk stratification
ACSQHC,^15^ 2009	Narrative	Any previous fall	NA	NA	List of assessment tool options	NA	Demographic risk factors
FSGG,^17^ 2011	Algorithm	1 Fall in 12 mo: gait and balance evaluation; recurrent falls in 12 mo or an acute fall or difficulty with walking and balance: fall evaluation^1^ and multifactorial intervention	≥80	Female	TUG test	Multiple risk factors^2^	Individuals with no fall history or low (1 fall) fall history may fall and require fall evaluation^36^
NICE,^18^ 2013	Narrative	≥1 Fall or emergency department visit for fall: multifactorial risk assessment	NA	NA	List of assessment tool options	Gait or balance problems	Broad list of assessment tools
STEADI,^27^ 2013	Algorithm	≥1 Fall in 12 mo: multifactorial risk assessment; low risk (no fall history): patient education and referral to community exercise, balance, fitness, or fall prevention program	NA	NA	TUG test, 4-stage balance test	Fear of falling	Demographic risk factors
Jung et al,^22^ 2014	Algorithm	≥1 Fall in 12 mo: comprehensive intervention; low risk (no fall history) regular checkups	NA	NA	Gait or balance problems	Polypharmacy, dementia, general diseases, cognition, fall-related symptoms, physical fitness, environmental factors, and aids	Details pertaining to assessment tools
RACGP,^26^ 2016	Table	≥1 Fall with multiple risk factors in 12 mo: risk factor screening and preventive activities; low risk (no fall history): yearly fall screening	≥65	NA	Gait or balance and mobility difficulties	Clinical judgment	Broad list of assessment tools
KAIM/KGS,^23^ 2017	Algorithm	≥2 Falls in 12 mo: multifactorial falls evaluation	NA	NA	TUG test and BBS	NA	Individuals with no fall history or low (1 fall) fall history may fall and require fall evaluation^36^
RNAO,^25^ 2017	Narrative	Any previous falls: comprehensive assessment; ≥2 falls in 12 mo: clinician referral	NA	NA	Gait, balance, or mobility difficulty	Clinical judgment	Broad list of assessment tools
SENATOR ONTOP,^20^ 2017	Narrative	Any previous falls: multifactorial intervention; low risk (no fall history): group-based exercise	≥65	NA	Gait problems	Walking aid, dizziness, muscle weakness, and polypharmacy	Unspecified assessments to determine risk
USPSTF,^14^ 2018	Table	Any previous falls: multifactorial intervention	≥65	NA	TUG test	Physical function or mobility problems	Adults aged 60-65 y may experience falls

Abbreviations: ACSQHC, Australian Commission on Safety and Quality in Health Care; AGILE, a recognized professional network of the Chartered Society of Physiotherapy; AGS/BGS/AAOS, American Geriatrics Society/British Geriatrics Society/American Academy of Orthopaedic Surgeons; BBS, Berg Balance Scale; FSGG, French Society of Geriatrics and Gerontology; KAIM/KGS, Korean Association of Internal Medicine/Korean Geriatrics Society; NA, not applicable; NICE, National Institute for Health and Care Excellence; RACGP, The Royal Australian College of General Practitioners; RNAO, Registered Nurses’ Association of Ontario; SENATOR ONTOP, software engine for the assessment & optimization of drug and non-drug therapy—older persons optimal evidence-based non-drug therapies in older people; STEADI, Stopping Elderly Accidents, Deaths and Injuries; TUG, Timed Up and Go; USPSTF, US Preventive Services Task Force.

For individuals who screened positive in fall history, several guidelines stratified their risk by demographic factors (ie, advanced age or female sex)^14,17,20,24,26^ or clinical characteristics (gait and balance abnormalities).^13,14,15,16,17,18,20,22,23,24,25,26,27^ The assessment of balance and gait, at this step, was recommended in 13 out of 15 guidelines^13,14,15,16,17,18,20,22,23,24,25,26,27^ using the Timed Up and Go Test (TUG),^37^ the Berg Balance Scale,^38^ and the Tinetti Performance-Oriented Mobility Assessment Tool,^39^ with the TUG being the most recommended test, appearing in 6 of the 15 guidelines (Table 3).^13,14,16,17,23,27^

## Discussion

This systematic review identified 15 high-quality practice guidelines for fall prevention and management, which provided 198 recommendations for risk assessment, prevention, and management of falls for older adults. Most guidelines strongly recommended risk stratification screening using short questionnaires and reserving gait and balance testing for those who screened positive. Similarly, most guidelines strongly recommended medication review, exercise interventions, environment modifications, multifactorial approaches, and active management of fractures and osteoporosis as key elements in the prevention of falls. Vision or footwear intervention, physiotherapy referral, and cardiovascular interventions were less commonly addressed. Although all selected guidelines had high overall methodologic quality, clinical applicability and stakeholder involvement were domains missed or lacking in details.

Recommendations for vitamin D supplementation showed mixed results. The strength of recommendations varied from strong to weak, with several guidelines not making any suggestions. This may reflect the inconsistent evidence about vitamin D supplementation for fall prevention and how the evidence varied based on settings: community vs residential or nursing home care.^40,41,42^ Similarly, recommendations about fall prevention education were mixed with a similar pattern seen. The use of hip protectors and digital technologies and wearables were often not included, with half of guidelines making no recommendation in these areas. The latest Cochrane meta-analyses found only weak evidence supporting the efficacy of hip protectors in preventing fractures after a fall in long-term care facilities^43^ and noted challenges in implementing the daily use of these protectors. This weak evidence, coupled with not all guidelines addressing falls in long-term care, likely explains the omission of hip protector recommendations.^44^ The underrepresentation of digital and wearable technologies is probably a reflection of their novelty.^45^

Risk stratification was an area addressed by most guidelines, with some proposing a specific risk stratification algorithm. Those algorithms often recommended performing gait and balance tests for individuals who screened positive.^38,39^ The most frequently recommended gait and balance test was the TUG, potentially owing to its simplicity, acceptance, and ease of administration. Evidence does not support acceptable predictive validity for any of the tests recommended in isolation for falls prediction,^46,47,48,49,50^ and specifically the TUG has low predictive validity.^51^ Consistent with the overall lower score in the applicability domain of the AGREE-II scale, details on resources, facilitators, and barriers to use of any of the recommended tests warranted more complete descriptions.

In addition to gait and balance, 5 guidelines stratified risk by some demographic characteristics (ie, advanced age, female sex).^14,17,20,24,26^ Explicit statements within the guidelines indicating the validation of their stratification algorithms were lacking. Few studies assessed the predictive accuracy of some of the proposed algorithms and found low sensitivity to detect individuals at higher risk of falls.^2,3,52^ Future guidelines providing risk stratification algorithms should conduct validation studies of their effectiveness, address the adaptability of the proposed algorithm to different care or residential settings, and include validations in resource-constrained areas, such as low- and middle-income countries. Finally, only 2 guidelines recommended active interventions with follow-up care for individuals deemed low risk in their stratification strategy, including education and exercises involving balance and lower limb strengthening.^20,22,26,27^ Recommendations for low-risk older adults that may help prevent falls and improve their overall health are also needed.

Recommendations to evaluate and manage medication-related risks for falls varied from judicious deprescribing of psychotropic and cardiovascular medications to performing a comprehensive medication review. Across all guidelines, medication review was recommended generally and in all settings. Although a search for medication review for fall risk–increasing drugs was identified in medication review recommendations, resources and tools for clinicians were lacking. This deficiency may have been attributable to the unavailability of resources. Tools such as STOPPFall (Screening Tool of Older Persons Prescriptions in older adults with high fall risk) have only recently been developed.^53^

Specific recommendations for older adults with cognitive impairment were scarce. Although cognitive evaluation was recommended as part of the initial assessment in most of the guidelines, specific guidance for evaluating specific aspects of cognition associated with increased fall risk (such as deficits in executive function) were lacking, despite the evidence in the literature of elevated fall risk factors in this group.^54,55^ Consideration of specific cognitive domains is imperative because executive function deficits are a known and prominent risk factor for falls among older adults—even among individuals without a formal diagnosis of cognitive impairment or dementia.^54,56,57,58^ Executive function may be a target for fall prevention interventions, as shown in recent studies.^59,60^ Future guidelines should consider including specific recommendations for individuals with cognitive deficits, including executive functioning and memory.^57,61^

The perspectives of people with a history of falls and associated injuries were not thoroughly and consistently embedded in the appraised guidelines. Moreover, personalized approaches that incorporate individual preferences in the fall prevention recommendations made to patients were also lacking.^62,63^ In general, patient and caregiver perspectives have not been consistently incorporated in clinical practice guidelines and related health resources.^64^

Clinical applicability was underrepresented in all the guidelines. Facilitators or barriers to implement recommendations were thoroughly detailed in only 3 guidelines.^15,25,27^ Similarly, advice or toolkits on how to implement the recommendations into practice were described in detail in only 5 guidelines,^15,18,21,24,25^ potential resource implications of applying the recommendations were detailed in only 2 guidelines,^15,18^ and monitoring or auditing criteria were discussed in only 2 guidelines.^18,25^ Our findings suggest that the challenges encountered in implementing recommendations should be better addressed in future clinical practice guidelines. A complementary way to address implementing recommendations is by following the example of the Stopping Elderly Accidents, Deaths and Injuries initiative from the Centers for Disease Control and Prevention, which focuses more on practical implementation with toolkits for the AGS/BGS/AAOS guidelines, as opposed to standing alone as a clinical practice guideline.

Finally, all the selected guidelines in our systematic review were led by authors from developed countries. We used 60 years of age or older as the definition of older adults to be geographically inclusive in our search^1^; however, our search found only a limited number of clinical practice guidelines from low- and middle-income countries, which were not evidence-based or based on a formal expert consensus process. This finding may reflect the lack of guidelines for fall prevention in many regions of the developing world, which may represent inadequate attention to this phenomenon or limited resources to develop clinical practice guidelines for older adults.

### Limitations

This systematic review has some limitations. Although no language restrictions were placed on our search, bibliographic databases of peer-reviewed papers included only journals that were indexed, which are mainly published in English. In addition, there is a possibility that we missed relevant clinical practice guidelines that were not in the databases searched, known to the experts we consulted, or on the public or health policy sites we examined.

## Conclusions

This systematic review found high agreement across clinical practice guidelines with strong recommendations for risk stratification, the use of specific tests for gait and balance assessments, multifactorial interventions, medication review, physical exercise, vision and footwear intervention, physiotherapy referral, environment modification, management of osteoporosis and fracture risk, and cardiovascular interventions. Recommendations on vitamin D supplementation and educational programs for fall prevention and management were inconsistent, whereas recommendations on hip protectors and wearable technologies were often not included. Future clinical practice guidelines should better address the clinical applicability of their recommendations, with more explicit consideration of resources, costs, and implementation barriers. Patients’ and caregivers’ perspectives should also be better reflected in developing future fall prevention and management guidelines for older adults. Our findings may assist clinicians in choosing the best-suited guidelines and recommendations for their setting and resource availability. The gaps detected may inform future guideline development, including the recent international initiative: World Falls Guidelines.^32,65^

## Notes

Supplement 1. **eTable 1.** Search Syntax **eTable 2.** Standardized Data Collection Form **eTable 3.** Glossary of Key Terms
eReferences.

Supplement 2.
Nonauthor Collaborators
