# Error in Supplement 2

**DOI:** 10.1001/jamanetworkopen.2023.32257

**Published:** 2023-08-28

In the Original Investigation titled “Evaluation of Clinical Practice Guidelines on Fall Prevention and Management for Older Adults: A Systematic Review,”^1^ published December 15, 2021, in Supplement 2, the surname of one of the members of the Task Force on Global Guidelines for Falls in Older Adults should be Ferrucci, not Ferruci. This article has been corrected.^1^
