# Supplementary material for: Evaluation of Clinical Practice Guidelines on Fall Prevention and Management for Older Adults: A Systematic Review
Source: JAMA Netw Open. 2021 Dec 15;4(12):e2138911. doi: 10.1001/jamanetworkopen.2021.38911 (PMC8674747; doi:10.1001/jamanetworkopen.2021.38911)
Supplement: Supplement 1. — eTable 1. Search Syntax eTable 2. Standardized Data Collection Form eTable 3. Glossary of Key Terms eReferences. [file jamanetwopen-e2138911-s001.pdf]

Supplementary Online Content

Montero-Odasso MM, Kamkar N, Pieruccini-Faria F, et al; Task Force on Global Guidelines for Falls in Older Adults. Evaluation of clinical practice guidelines on fall prevention and management for older adults: a systematic review. *JAMA Netw Open*. 2021;4(12):e2138911. doi:10.1001/jamanetworkopen.2021.38911

eTable 1. Search Syntax
eTable 2. Standardized Data Collection Form
eTable 3. Glossary of Key Terms
eReferences.

This supplementary material has been provided by the authors to give readers additional information about their work.

© 2021 Montero-Odasso MM et al. *JAMA Network Open*.

eTable 1. Search Syntax

Search Concept	Search Line #	Search Strategy
**Falls**	1	Accidental Falls/
	2	(fall or falls or falling or fallers).ti,ab,kw,kf.
	3	(fall* adj2 fracture*).ti,ab,kf,kw.
	**4**	**1 or 2 or 3**
**Clinical Practice Guideline**	5	"best practice guideline*".ti,ab,kf,kw.
	6	strateg*.ti,ab,kf,kw.
	7	"WHO Global Report*".ti,ab,kf,kw.
	8	exp clinical pathway/
	9	exp clinical protocol/
	10	exp consensus/
	11	exp consensus development conference/
	12	exp consensus development conferences as topic/
	13	critical pathways/
	14	exp guideline/
	15	guidelines as topic/
	16	exp practice guideline/
	17	practice guidelines as topic/
	18	health planning guidelines/
	19	(guideline or practice guideline or consensus development conference or consensus development conference, NIH).pt.
	20	(position statement* or policy statement* or practice parameter* or best practice*).ti,ab,kf,kw.
	21	(standards or guideline or guidelines).ti,ab,kw,kf.
	22	((practice or treatment* or clinical) adj guideline*).ti,ab,kw,kf.
	23	(CPG or CPGs).ti,ab,kf,kw.
	24	consensus*.ti,kf,kw.
	25	consensus*.ab. /freq=2
	26	((critical or clinical or practice) adj2 (path or paths or pathway or pathways or protocol*)).ti,ab,kf,kw.
	27	recommendat*.ti,ab,kf,kw.
	28	(care adj2 (standard or path or paths or pathway or pathways or map or maps or plan or plans)).ti,ab,kf,kw.
	29	(algorithm* adj2 (screening or examination or test or tested or testing or assessment* or diagnosis or diagnoses or diagnosed or diagnosing)).ti,ab,kf,kw.
	30	(algorithm* adj2 (pharmacotherap* or chemotherap* or chemotreatment* or therap* or treatment* or intervention*)).ti,ab,kf,kw.
	**31**	**or/5-30**
**Management and Prevention**	32	Accident Prevention/
	33	prevent*.ti,kf,kw.
	34	manage*.ti,ab,kf,kw.

© 2021 Montero-Odasso MM et al. *JAMA Network Open*.

	35	prevent.ab. /freq=2
	36	"prevention and control".ti,ab,kf,kw.
	37	(prevent* and control*).ti,ab,kf,kw.
	38	(prevent* or control*).ti,ab,kf,kw.
	**39**	**or/32-38**
**Older Adults**	40	Geriatric.mp. or exp Geriatrics/
	41	Gerontology.mp. or Geriatrics/
	42	(old* adj2 (adult or person or people or age*)).ti,ab,kf,kw.
	43	senior.mp. or exp Senior Centers/
	**44**	**or/40-43**
**Total**	45	4 and 31 and 39 and 44

© 2021 Montero-Odasso MM et al. *JAMA Network Open*.

eTable 2. Standardized Data Collection Form

Information to Extract	Definitions and Details	Example Extraction
General Study Information		
Extractor Initials	Your initials	*N.K.*
Author	First author of the guideline.	*Smith et al.*
Study ID	Numerical Identifier for guideline.	*#1*
Year	Year of publication.	*2010*
Title	Title of guideline published.	*Preventing falls*
Organization	Organization or society supporting guideline (if applicable).	*ABC Society*
Inclusion Criteria		
i) Falls outcome	Purpose of guideline is fall reduction, prevention and/or management.	*Yes*
ii) Study type	Clinical practice guidelines for preventing and/or managing falls categorized as consensus and/or evidence- based guidelines (S2 or S3 classification from the Association of the Scientific Medical Societies (AWMF) Assessment of clinical practice guidelines.	*Yes*
iii) Target population of guidelines	Older adults 60 years or older.	*Yes*
AGREE-II Quality Assessment (scores range from 1-7 on 23 items)		
AGREE-II Item 1.	The overall objective(s) of the guideline is (are) specifically described	*7*
AGREE-II Item 2.	The health question(s) covered by the guideline is (are) specifically described.	*5*
AGREE-II Item 3.	The population (patients, public, etc.) to whom the guideline is meant to apply is specifically described.	*6*
AGREE-II Item 4.	The guideline development group includes individuals from all relevant professional groups.	*7*
AGREE-II Item 5.	The views and preferences of the target population (patients, public, etc.) have been sought.	*5*
AGREE-II Item 6.	The target users of the guideline are clearly defined.	*5*
AGREE-II Item 7.	Systematic methods were used to search for evidence.	*4*
AGREE-II Item 8.	The criteria for selecting the evidence are clearly described.	*7*
AGREE-II Item 9.	The strengths and limitations of the body of evidence are clearly described.	*7*
AGREE-II Item 10.	The methods for formulating the recommendations are clearly described.	*6*
AGREE-II Item 11.	The health benefits, side effects, and risks have been considered in formulating the recommendations.	*4*
AGREE-II Item 12.	There is an explicit link between the recommendations and the supporting evidence.	*4*
AGREE-II Item 13.	The guideline has been externally reviewed by experts prior to its publication.	*6*
AGREE-II Item 14.	A procedure for updating the guideline is provided.	*7*
AGREE-II Item 15.	The recommendations are specific and unambiguous.	*5*
AGREE-II Item 16.	The different options for management of the condition or health issue are clearly presented.	*5*
AGREE-II Item 17.	Key recommendations are easily identifiable.	*4*
AGREE-II Item 18.	The guideline describes facilitators and barriers to its application.	*7*

© 2021 Montero-Odasso MM et al. *JAMA Network Open*.

AGREE-II Item 19.	The guideline provides advice and/or tools on how the recommendations can be put into practice.	*5*
AGREE-II Item 20.	The potential resource implications of applying the recommendations have been considered.	*5*
AGREE-II Item 21.	The guideline presents monitoring and/or auditing criteria.	*6*
AGREE-II Item 22.	The views of the funding body have not influenced the content of the guideline.	*7*
AGREE-II Item 23.	Competing interests of guideline development group members have been recorded and addressed.	*7*
GRADE Recommendations Across Areas<br>*Strength of Recommendation: 1 = strong; 2 = weak.*<br>*Quality of Evidence: A = high quality B = moderate quality; C = low quality.*		
Risk Stratification	Recommendations for risk stratification include any method specified to stratify individuals who are at a high, moderate, or low risk for future falls.	*1A*
Assessment Tools	Recommendations on the use of assessment tools refer to any advice or guidance on specific fall risk assessment tools including balance, gait, and/or mobility assessment tools.	*1B*
Fractures and Osteoporosis Management	In the present review, recommendations related to fractures and osteoporosis management include any advice or guidance to prevent or treat fractures and/or osteoporosis as risk factors for falls and fall-related injuries.	*1B*
Multifactorial Interventions	Recommendations for multifactorial interventions include any recommendation to perform several intervention strategies simultaneously (i.e., physical exercise paired with vision modification) to prevent or minimize falls and related injuries.	*1A*
Medication Review	A systematic assessment of medications from medical records, prescriptions, and supplementation by qualified medical personnel such as a physician, nurse, or pharmacist.	*1C*
Exercise Interventions	Recommendations for exercise include any guidance or advice to perform physical activity and exercise to prevent future falls and/or manage consequences of past falls.	*1A*
Vitamin D Supplements	In the present review, recommendations in vitamin D entail any guidance or advice on supplementation with vitamin D to prevent and/or manage falls.	*2C*
Hip Protectors	Recommendations for the use of hip protectors include any. guidance on the use of hip protectors for the prevention of fall-related injuries. Hip protectors consist of foam pads or plastic shields that are worn to protect and shield individuals from hip fractures following a fall.	*N/A*
Vision Modification	Recommendations pertaining to vision modification consist of any advice or guidance to correct visual impairment for the prevention of future falls. Vision modification methods include the prescription of glasses and/or contact lenses, as well as cataract surgery to correct for visual impairment.	*1A*
Environment Modification	Environment modification recommendations consist of any guidance or advice to modify the individual's home environment to prevent obstacles that may increase the risk of falls. Environment modification also includes advice to prevent slips by modifying flooring to provide better grip and reduce loose rugs and carpets, which may be tripping hazards.	*1A*
Cognitive Factors	Recommendations on cognitive factors and their management	*1B*

© 2021 Montero-Odasso MM et al. *JAMA Network Open*.

Management	include any recognition of cognitive risk factors for falls, including evaluation of cognitive impairment, performance in specific cognitive domains, and/or cognitive management with interventions to enhance cognitive function and potentially reduce fall risk.	
Physiotherapy Referral	Recommendations on physiotherapy referral include any advice for the individual to seek help from a physiotherapist to prevent future falls and/or manage symptoms of previous falls.	*1C*
Falls Education	Recommendations on falls education include any advice or guidance for individuals to receive education on fall prevention and management.	*N/A*
Cardiovascular Interventions	Recommendations for cardiovascular interventions include any advice or guidance geared towards management of cardiovascular risk factors for falls: this includes recommendations for individuals with cardiovascular conditions (i.e., blood pressure outside of age/sex typical norms).	*1B*
Footwear Evaluation and Intervention	Recommendations related to footwear include any advice or guidance to modify footwear to reduce slips, trips, and falls.	*1B*
Technology	Any technology to prevent and/or manage falls, including wearable technological devices that can be worn on the individual while moving (i.e., watch) and communicate attributes of the individual to her/him based on sensors (i.e., step-count).	*N/A*
	Risk Stratification	
Algorithm	Did the guideline include an algorithm to stratify risk as high, moderate, or low?	*Yes*
Presentation Type	How did the guideline present the risk stratification? (i.e., as a table, algorithm, or text).	*Table*
Algorithm Validated	Was the algorithm (if present) validated for use?	*No*
Fall History	Did the risk stratification method include fall history as a risk factor for falls?	*Yes*
Age/Sex	Did the risk stratification method include age and/or sex as risk factors for falls?	*No*
Gait/Balance/Mobility Assessment	Did the risk stratification method include assessment of gait, balance, or mobility to determine fall risk?	*Yes*
Name of Assessment	If the risk stratification method included assessment of gait, balance, or mobility, what assessment tool was recommended?	*TUG*
Other	Were there any other risk stratification methods to determine falls risk? If so, please indicate.	*Yes, fear of falling.*

© 2021 Montero-Odasso MM et al. *JAMA Network Open*.

eTable 3. Glossary of Key Terms

GLOSSARY OF KEY TERMS	
**AGREE-II**	The Appraisal of Guidelines for REsearch & Evaluation (AGREE) Instrument is a tool that assesses the methodological rigour and transparency in which a guideline is developed.[1]
**Assessment Tools**	In the present review, recommendations on the use of assessment tools refer to any advice or guidance on specific fall risk assessment tools including balance, gait, and/or mobility assessment tools.
**Cardiovascular Intervention**	In the present review, recommendations for cardiovascular interventions include any advice or guidance geared towards management of syncope and cardiovascular risk factors for falls: this includes recommendations for individuals with cardiovascular conditions (i.e., blood pressure outside of age/sex typical norms).
**Caregiver**	In the present review, caregivers or carers encompass all individuals who care for older adults.
**Clinical Practice Guideline**	Recommendations on diagnosis and treatment of a medical condition predominantly for healthcare professionals to use in clinical practice.[2]
**Cognitive Factors and Management**	In the present review, recommendations on cognitive factors and their management include any recognition of cognitive risk factors for falls, including evaluation of cognitive impairment, performance in specific cognitive domains, and/or cognitive management with interventions to enhance cognitive function and potentially reduce fall risk.
**Deprescribing**	The process of withdrawal of an inappropriate medication under the supervision of a health care professional to manage polypharmacy and improving outcomes.[3]
**Environment Modification**	In the present review, environment modification recommendations consist of any guidance or advice to modify the individual's home environment to prevent obstacles that may increase the risk of falls. Environment modification also includes advice to prevent slips by modifying flooring to provide better grip and reduce loose rugs and carpets, which may be tripping hazards.
**Exercise**	In the present review, recommendations for exercise include any guidance or advice to perform structured physical activity to prevent future falls and/or manage consequences of past falls.
**Fall**	An event in which an individual comes to rest on the ground, floor, or lower level.[4]
**Fall Related Injury**	An injury sustained following a fall.[5]
**Fall Risk Assessment**	A set of assessments performed to identify individuals at highest risk for falls, upon whom to target specific interventions.[6]
**Fall Risk Increasing Drugs (FRIDs)**	Medications known to increase the risk of falls.[7-10]
**Falls Education**	In the present review, recommendations on falls education include any advice or guidance for individuals to receive education on fall prevention and management.
**Fall Risk Stratification Algorithm**	The systematic process of decision-making and intervention that should occur for falls risk case findings in patients.[11]
**Footwear Evaluation and Intervention**	In the present review, recommendations related to footwear include any advice or guidance to modify footwear to reduce slips, trips, and falls.

**Fractures and Osteoporosis Management**	In the present review, recommendations related to fractures and osteoporosis management include any advice or guidance to prevent, or treat fractures and/or osteoporosis as risk factors for falls and fall-related injuries.
**GRADE Recommendations**	The ‘‘Grades of Recommendation, Assessment, Development, and Evaluation’’ (GRADE) approach provides guidance for rating quality of evidence and grading strength of recommendations in health care.[12]
**Hip Protectors**	In the present review, recommendations for the use of hip protectors include any. guidance on the use of hip protectors for the prevention of fall-related injuries. Hip protectors consist of foam pads or plastic shields that are worn to protect and shield individuals from hip fractures following a fall.[13]
**Medication Review**	A systematic assessment of medications from medical records, prescriptions, and supplementation by qualified medical personnel such as a physician, nurse, or pharmacist.[14]
**Multifactorial Interventions**	In the present review, recommendations for multifactorial interventions include any recommendation to perform several intervention strategies simultaneously (i.e., physical exercise paired with vision modification) to prevent or minimize falls and related injuries.
**Older Adults**	Individuals 60 years of age or older.[15,16]
**Physiotherapy Referral**	In the present review, recommendations on physiotherapy referral include any advice for the individual to seek help from a physiotherapist to prevent future falls and/or manage symptoms of previous falls.
**Risk Stratification**	In the present review, recommendations for risk stratification include any method specified to stratify individuals who are at a high, moderate, or low risk for future falls.
**Stakeholders**	In the present review, stakeholders encompass all individuals who may be concerned with falls and their prevention. This includes clinicians providing medical services to older adults at risk for falls, as well as the older adult patients themselves, and any caregivers (such as family members or friends) who care for older adults.[17]
**STOPPFall**	A screening tool used to identify pharmacological prescriptions that may increase the risk of falls in older adults.[18]
**Technology (including wearables)**	Any technology to prevent and/or manage falls, including wearable technological devices that can be worn on the individual while moving (i.e., watch) and communicate attributes of the individual to her/him based on sensors (i.e., step-count).[19]
**Vitamin D**	In the present review, recommendations in vitamin D entail any guidance or advice on supplementation with vitamin D to prevent and/or manage falls.
**Vision Modification**	In the present review, recommendations pertaining to vision modification consist of any advice or guidance to correct visual impairment for the prevention of future falls. Vision modification methods include the prescription of glasses and/or contact lenses, as well as cataract surgery to correct for visual impairment.

© 2021 Montero-Odasso MM et al. *JAMA Network Open*.

eReferences.

1. Brouwers MC, Kho ME, Browman GP, et al. AGREE II: advancing guideline development, reporting, and evaluation in health care. *Prev Med.* 2010;51(5):421-424.
2. IQWiG. *What are clinical practice guidelines?* Cologne, Germany: *Institute for Quality and Efficiency in Health Care*;2016.
3. Reeve E, Gnjidic D, Long J, Hilmer S. A systematic review of the emerging de fi nition of 'deprescribing' with network analysis: implications for future research and clinical practice. *Br J Clin Pharmacol.* 2015;80(6):1254-1268.
4. Lamb SE, Jorstad-Stein EC, Hauer K, Becker C, Prevention of Falls Network E, Outcomes Consensus G. Development of a common outcome data set for fall injury prevention trials: the Prevention of Falls Network Europe consensus. *J Am Geriatr Soc.* 2005;53(9):1618-1622.
5. Oliver D, Healey F, Haines TP. Preventing falls and fall-related injuries in hospitals. *Clin Geriatr Med.* 2010;26(4):645-692.
6. Perell KL, Nelson A, Goldman RL, Luther SL, Prieto-Lewis N, Rubenstein LZ. Fall risk assessment measures: an analytic review. *J Gerontol A Biol Sci Med Sci.* 2001;56(12):M761-766.
7. Seppala LJ, van de Glind EMM, Daams JG, et al. Fall-Risk-Increasing Drugs: A Systematic Review and Meta-analysis: III. Others. *J Am Med Dir Assoc.* 2018;19(4):372 e371-372 e378.
8. Seppala LJ, van der Velde N, Masud T, et al. EuGMS Task and Finish group on Fall-Risk-Increasing Drugs (FRIDs): Position on Knowledge Dissemination, Management, and Future Research. *Drugs Aging.* 2019;36(4):299-307.
9. de Vries M, Seppala LJ, Daams JG, et al. Fall-Risk-Increasing Drugs: A Systematic Review and Meta-Analysis: I. Cardiovascular Drugs. *J Am Med Dir Assoc.* 2018;19(4):371 e371-371 e379.
10. Seppala LJ, Wermelink A, de Vries M, et al. Fall-Risk-Increasing Drugs: A Systematic Review and Meta-Analysis: II. Psychotropics. *J Am Med Dir Assoc.* 2018;19(4):371 e311-371 e317.
11. Guideline for the prevention of falls in older persons. American Geriatrics Society, British Geriatrics Society, and American Academy of Orthopaedic Surgeons Panel on Falls Prevention. *J Am Geriatr Soc.* 2001;49(5):664-672.
12. Guyatt GH, Oxman AD, Schunemann HJ, Tugwell P, Knottnerus A. GRADE guidelines: a new series of articles in the Journal of Clinical Epidemiology. *J Clin Epidemiol.* 2011;64(4):380-382.
13. Santesso N, Carrasco-Labra A, Brignardello-Petersen R. Hip protectors for preventing hip fractures in older people. *Cochrane Database Syst Rev.* 2014(3):CD001255.
14. Sjoberg C, Wallerstedt SM. Effects of medication reviews performed by a physician on treatment with fracture-preventing and fall-risk-increasing drugs in older adults with hip fracture-a randomized controlled study. *J Am Geriatr Soc.* 2013;61(9):1464-1472.
15. Snider EL. Young-old versus old-old and the use of health services. Does the difference make a difference? *J Am Geriatr Soc.* 1981;29(8):354-358.
16. World Health Organization: Ageing LCU. *WHO global report on falls prevention in older age.* WHO; 2008.
17. Laing SS, Silver IF, York S, Phelan EA. Fall prevention knowledge, attitude, and practices of community stakeholders and older adults. *J Aging Res.* 2011;2011:395357.
18. Seppala LJ, Petrovic M, Ryg J, et al. STOPPFall (Screening Tool of Older Persons Prescriptions in older adults with high fall risk): a Delphi study by the EuGMS Task and Finish Group on Fall-Risk-Increasing Drugs. *Age Ageing.* 2020;50(4):189-1199.
19. Godfrey A. Wearables for independent living in older adults: Gait and falls. *Maturitas.* 2017;100:16-26.

© 2021 Montero-Odasso MM et al. *JAMA Network Open*.
