# Supplementary material for: Evaluation of Clinical Practice Guidelines on Fall Prevention and Management for Older Adults: A Systematic Review
Source: JAMA Netw Open. 2021 Dec 15;4(12):e2138911. doi: 10.1001/jamanetworkopen.2021.38911 (PMC8674747; doi:10.1001/jamanetworkopen.2021.38911)
Supplement: Supplement 2. — Nonauthor Collaborators [file jamanetwopen-e2138911-s002.pdf]

*Indicates required information. Only first name, last name, and suffix will appear in PubMed.

*Group Name(s): Task Force on Global Guidelines for Falls in Older Adults							
***First Name and Middle Initial(s)**	***Last Name**	***Suffix (eg, Jr, III)**	Academic Degrees	Institution	Location (city, state/province, country)	Role or Contribution, eg, chair, principal investigator	Group (if more than 1 Group listed in the byline) and/or Subgroup (eg, Steering Committee)
Mirko	Petrovic				Belgium	Steering Committee Member, Working Group Leader, and World Expert	Working Group 2
Alice	Nieuwboer				Belgium	Working Group Leader and World Expert	Working Group 7
Ellen	Vlaeyen				Belgium	Working Group Member and World Expert	Working Group 5
Koen	Milisen				Belgium	Steering Committee Member, Working Group Leader, and World Expert	Working Group 5
Jesper	Ryg				Denmark	Steering Committee Member, Working Group Leader, and World Expert	Working Group 1
Rose Anne	Kenny				Ireland	Steering Committee Member, Working Group Leader, and World Expert	Working Group 3
Robert	Bourke				Ireland	Working Group Member and World Expert	Working Group 3
Sirpa	Hartikainen				Finland	Working Group Member and World Expert	Working Group 2

*Indicates required information. Only first name, last name, and suffix will appear in PubMed.

***First Name and Middle Initial(s)**	***Last Name**	***Suffix (eg, Jr, III)**	Academic Degrees	Institution	Location (city, state/province, country)	Role or Contribution, eg, chair, principal investigator	Group (if more than 1 Group listed in the byline) and/or Subgroup (eg, Steering Committee)
Tischa	Van der Cammen				Netherlands	Working Group Leader and World Expert	Working Group 8
Nathalie	Van der Velde				Netherlands	Co-Chair, Steering Committee Member, Working Group Leader, and World Expert	Working Group 2 and Working Group 10
Tahir	Masud				United Kingdom	Co-Chair, Steering Committee Member, Working Group Leader, and World Expert	Working Group 1 and Working Group 10
Chris	Todd				United Kingdom	World Expert	
Finbarr C.	Martin				United Kingdom	Steering Committee Member and World Expert	
David R.	Marsh				United Kingdom	Steering Committee Member and World Expert	
Sallie	Lamb				United Kingdom	Steering Committee Member and World Expert	
James	Frith				United Kingdom	World Expert	
Pip	Logan				United Kingdom	Steering Committee Member, Working Group Leader, and World Expert	Working Group 10
Dawn	Skelton				United Kingdom	Steering Committee Member, Working Group Leader, and World Expert	Working Group 4

Supplemental Online Content: Nonauthor Collaborators

*Indicates required information. Only first name, last name, and suffix will appear in PubMed.

*First Name and Middle Initial(s)	*Last Name	*Suffix (eg, Jr, III)	Academic Degrees	Institution	Location (city, state/province, country)	Role or Contribution, eg, chair, principal investigator	Group (if more than 1 Group listed in the byline) and/or Subgroup (eg, Steering Committee)
Hubert	Blain				France	Steering Committee Member and World Expert	
Cedric	Anweiller				France	World Expert	
Ellen	Freiberger				Germany	Working Group Leader and World Expert	Working Group 1
Clemens	Becker				Germany	Steering Committee Member, Working Group Leader, and World Expert	Working Group 8
Matteo	Cesari				Italy	World Expert	
Alvaro	Casas-Herrero				Spain	World Expert	
Javier Perez	Jara				Spain	World Expert	
Christina Alonzo	Bouzòn				Spain	World Expert	
Ana-Karim	Welmer				Sweden	World Expert	
Stephanie	Birnghebuam				Switzerland	World Expert	
Reto	Kressig				Switzerland	World Expert	
Manuel	Montero-Odasso				Canada	Chair, Steering Committee Member, Working Group Leader, and World Expert	Working Group 6 and Working Group 10
Mark	Speechley				Canada	Working Group Leader and World Expert	Working Group 10
Bill	McIlroy				Canada	World Expert	
Susan	Hunter				Canada	Steering Committee Member, Working Group Leader, and World Expert	Working Group 6

*Indicates required information. Only first name, last name, and suffix will appear in PubMed.

***First Name and Middle Initial(s)**	***Last Name**	***Suffix (eg, Jr, III)**	Academic Degrees	Institution	Location (city, state/province, country)	Role or Contribution, eg, chair, principal investigator	Group (if more than 1 Group listed in the byline) and/or Subgroup (eg, Steering Committee)
Richard	Camicioli				Canada	Working Group Leader and World Expert	Working Group 7
Kenneth	Madden				Canada	World Expert	
Mireille	Norris				Canada	World Expert	
Jennifer	Watt				Canada	Working Group Leader and World Expert	Working Group 10
Louise	Mallet				Canada	Working Group Leader and World Expert	Working Group 2
David B.	Hogan				Canada	Steering Committee Member, Working Group Leader, and World Expert	Working Group 11: Cross Cutting Theme
Joe	Verghese				United States of America	Steering Committee Member, Working Group Leader, and World Expert	Working Group 6
Ervin	Sejdic				United States of America	Working Group Leader and World Expert	Working Group 8
Luigi	Ferruci				United States of America	World Expert	
Lewis	Lipsitz				United States of America	Steering Committee Member, Working Group Leader, and World Expert	Working Group 3
David A.	Ganz				United States of America	Steering Committee Member and World Expert	

*Indicates required information. Only first name, last name, and suffix will appear in PubMed.

*First Name and Middle Initial(s)	*Last Name	*Suffix (eg, Jr, III)	Academic Degrees	Institution	Location (city, state/province, country)	Role or Contribution, eg, chair, principal investigator	Group (if more than 1 Group listed in the byline) and/or Subgroup (eg, Steering Committee)
Neil B.	Alexander				United States of America	Working Group Leader and World Expert	Working Group 6
Fabiana	Giber				Argentina	World Expert	
Marcelo	Schapira				Argentina	World Expert	
Ricardo	Jauregui				Argentina	Steering Committee Member and World Expert	
Felipe	Melgar-Cuellar				Bolivia	World Expert	
Daniela Cristina	Carvalho de Abreu				Brazil	World Expert	
Roberto Alves	Lourenço				Brazil	World Expert	
Monica	Pierrucini				Brazil	Working Group Leader and World Expert	Working Group 9
Alejandro	Ceriani				Chile	World Expert	
Pedro	Marín-Larraín				Chile	World Expert	
Homero	Gac Espinola				Chile	World Expert	
José Fernando	Gómez-Montes				Colombia	Steering Committee Member, Working Group Leader, and World Expert	Working Group 9
Carlos Alberto	Cano-Gutierrez				Colombia	World Expert	
Xinia	Ramirez Ulate				Costa Rica	World Expert	
José Ernesto	Picado Ovares				Costa Rica	World Expert	
Patricio Gabriel	Buendia				Ecuador	World Expert	
Susana Lucia	Tito				Ecuador	World Expert	
Diego	Martínez Padilla				Ecuador	World Expert	
Sara G.	Aguilar-Navarro				Mexico	Working Group Member and World Expert	Working Group 10

*Indicates required information. Only first name, last name, and suffix will appear in PubMed.

***First Name and Middle Initial(s)**	***Last Name**	***Suffix (eg, Jr, III)**	Academic Degrees	Institution	Location (city, state/province, country)	Role or Contribution, eg, chair, principal investigator	Group (if more than 1 Group listed in the byline) and/or Subgroup (eg, Steering Committee)
Alberto	Mimenza				Mexico	Working Group Member and World Expert	Working Group 10
Rogelio	Moctezum				Mexico	Working Group Member and World Expert	Working Group 10
Alberto	Avila-Funes				Mexico	World Expert	
Luis Miguel	Gutiérrez-Robledo				Mexico	World Expert	
Luis Manuel	Cornejo Alemán				Panama	World Expert	
Edgar	Aguilera Caona				Paraguay	World Expert	
Juan Carlos	Carbajal				Peru	Working Group Member and World Expert	Working Group 10
José F.	Parodi				Peru	World Expert	
Aldo	Sgaravatti				Uruguay	World Expert	
Stephen	Lord				Australia	Steering Committee Member, Working Group Leader, and World Expert	Working Group 4
Cathie	Sherrington				Australia	Steering Committee Member, Working Group Leader, and World Expert	Working Group 4
Cathy	Said				Australia	Working Group Leader, and World Expert	Working Group 5
Ian	Cameron				Australia	Working Group Leader and World Expert	Working Group 10
Meg	Morris				Australia	Working Group Member and World Expert	Working Group 5

*Indicates required information. Only first name, last name, and suffix will appear in PubMed.

***First Name and Middle Initial(s)**	***Last Name**	***Suffix (eg, Jr, III)**	Academic Degrees	Institution	Location (city, state/province, country)	Role or Contribution, eg, chair, principal investigator	Group (if more than 1 Group listed in the byline) and/or Subgroup (eg, Steering Committee)
Gustavo	Duque				Australia	Working Group Leader and World Expert	Working Group 5
Jacqueline	Close				Australia	Steering Committee Member and World Expert	
Ngaire	Kerse				New Zealand	World Expert	
Maw Pin	Tan				Malaysia	Steering Committee Member, Working Group Leader, and World Expert	Working Group 9
Leilei	Duan				China	Steering Committee Member and World Expert	
Ryota	Sakurai				Japan	World Expert	
Chek	Hooi Wong				Singapore	World Expert	
Hossein	Negahban				Iran	World Expert	
Chang	Won Won				Korea	World Expert	
Jeffrey	Hausdorff				Israel	Working Group Leader and World Expert	Working Group 7 and Working Group 8
Sebastiana	Kalula				South Africa	World Expert	
Olive	Kobusingye				Uganda	World Expert	
